# Homogeneity and variation of donor doping in Verneuil-grown SrTiO_3_:Nb single crystals

**DOI:** 10.1038/srep32250

**Published:** 2016-08-31

**Authors:** C. Rodenbücher, M. Luysberg, A. Schwedt, V. Havel, F. Gunkel, J. Mayer, R. Waser

**Affiliations:** 1 Forschungszentrum Jülich GmbH, Peter Grünberg Institute (PGI-7) and JARA-FIT, 52425 Jülich, Germany; 2 Forschungszentrum Jülich GmbH, Ernst Ruska-Centre (ER-C) for Microscopy and Spectroscopy with Electrons, 52425 Jülich, Germany; 3 RWTH Aachen, Gemeinschaftslabor für Elektronenmikroskopie, 52056 Aachen, Germany; 4 RWTH Aachen University, Institute of Electronic Materials (IWE2), 52056 Aachen, Germany

## Abstract

The homogeneity of Verneuil-grown SrTiO_3_:Nb crystals was investigated. Due to the fast crystal growth process, inhomogeneities in the donor dopant distribution and variation in the dislocation density are expected to occur. In fact, for some crystals optical studies show variations in the density of Ti^3+^ states on the microscale and a cluster-like surface conductivity was reported in tip-induced resistive switching studies. However, our investigations by TEM, EDX mapping, and 3D atom probe reveal that the Nb donors are distributed in a statistically random manner, indicating that there is clearly no inhomogeneity on the macro-, micro-, and nanoscale in high quality Verneuil-grown crystals. In consequence, the electronic transport in the bulk of donor-doped crystals is homogeneous and it is not significantly channelled by extended defects such as dislocations which justifies using this material, for example, as electronically conducting substrate for epitaxial oxide film growth.

For many years the prototype transition metal oxide SrTiO_3_ has attracted enormous attention in the scientific community due to its unusual electronic properties. By appropriate doping it can change its electronic transport behaviour from insulating to metallic and even to superconducting^1^ making it a promising material for various future energy-efficient applications such as fuel cells^2^, solar cells^3^, sensors^4^, batteries^5^ and novel electronic devices^6^. In particular the potential of the resistive switching effect^7^,^8^,^9^ in SrTiO_3_ for building redox-based random access memories (ReRAM) and novel synaptic logic circuits has been investigated intensively and was documented by numerous publications. Furthermore, SrTiO_3_ doped with the donor Nb is the most popular conducting substrate for the epitaxial growth of functional oxide thin films. Pentavalent Nb substitutes the tetravalent Ti in the lattice upon doping introducing an additional charge that is compensated under reducing conditions by electrons in the conduction band leading to a degenerate, metal-like semiconductor^10^,^11^,^12^.

SrTiO_3_ shows a cubic perovskite structure ABO_3_ and has a Goldschmidt tolerance factor very close to 1 which–in the simple ionic picture - stabilizes the lattice against lattice distortions such as tilting of the TiO_6_ octahedra. Still, one might ask if at higher donor concentrations defect ordering by shear planes or by microdomains of different composition may occur as, for example, in the Sr/Ca distribution of the brownmillerite (Sr,Ca)FeO_2.5_^13^. In Nb-doped SrTiO_3_ ceramics, a certain segregation of Nb dopant ions at grain boundaries is observed^14^,^15^. Epitaxial thin films grown by pulsed laser deposition at relatively low temperatures often show cluster-like distortions of the perovskite lattice. In some cases, a correlation with inhomogeneities of the Nb concentration^16^ or the Nb valence^17^ has been reported, while in other cases no such correlation was observed^18^.

In single crystals, deviations from the statistically random distribution of dopant ions such as Nb in Sr(Ti,Nb)O_3_ solid solutions may also occur due to imperfections during crystal growth. Single crystals which are commercially available today are prepared by the Verneuil method which is a very cost-effective and fast method with a growth speed^19^ of typically 5∙10^4^ Å/s. It is well known that, in consequence, Verneuil-grown single crystals exhibit a large amount of extended defects such as dislocations with densities in the order of 10^5^–10^9^/cm^2^, compositional fluctuations, as well as striations related to internal stress fields^19^,^20^,^21^. While Scheel *et al*. conducted intense research on the influence of growth parameters on the crystal quality using the Verneuil and boron flux method and Belruss *et al*. succeeded in growing SrTiO_3_ crystals with high perfection using top-seeded solution growth in the 1970’s^19^,^22^,^23^, nowadays there are no companies on the market offering this kind of perfect crystals. Hence, nearly all publications dealing with doped SrTiO_3_ were based on Verneuil-grown crystals, but a fundamental investigation of crystal quality in particular with respect to donor distributions in the bulk of the crystal lattice and at dislocations that could potentially influence the electronic properties has not been conducted so far. Regarding the entropy of the ensemble of point defects at the high temperatures of crystal growth, classically a statistically random distribution of donors in the matrix would be expected on the macro-, micro-, and nanoscale. But since the Verneuil growth takes place far away from the thermodynamic equilibrium, a deviation from a statistical distribution cannot be excluded theoretically. In fact, we recently investigated the optical properties of Nb-doped SrTiO_3_ single crystals qualitatively using optical microscopy, confocal Raman microscopy, and fluorescence lifetime imaging microscopy revealing an inhomogeneous structure of the distribution of Ti^3+^ states on the microscale^24^ in randomly selected regions of several commercial crystals^25^. Furthermore, we found conductive clusters in the range of few tens of nanometres on the surface when we used local conductivity atomic force microscopy to perform redox-based resistive switching and it was speculated that these clusters may originate from inhomogeneous Nb distributions^26^. However, it remained unclear, if these inhomogeneities are really related to an inhomogeneous Nb distribution on the nanoscale and to what extent this finding is relevant for applications.

In this paper, we aim to objectify this question for Nb-doped SrTiO_3_ single crystals by performing quantitative investigations of the Nb distribution on the nanoscale by transmission electron microscopy (TEM) and energy dispersive X-ray (EDX) mapping. In addition, we study the impact of dislocations, since it was found that in undoped SrTiO_3_ dislocations play a key role for resistive switching phenomena by providing a “template” for the evolution of switchable filaments^8^,^27^. In the framework of classical lattice disorder with point defects we discuss the influence of local variations of the Nb concentration on the conductivity of the material and we consider the suitability of the material for building electronic devices.

## Results and Discussion

Verneuil-grown SrTi_1-*x*_Nb_*x*_O_3_ crystals with a concentration of *x* = 0.2 at%, 1 at%, 1.4 at% and 10.2 at% were purchased from Crystec, Mateck, and SurfaceNet with typical sizes of 5 mm × 5 mm × 0.5 mm and 10 mm × 10 mm × 0.5 mm. The cutting of the samples of the crystal boule, the polishing procedures, and the standard quality analysis have been performed by the producer. A photograph of a crystal is shown in the Supplementary Information I. Here, we present detailed investigations of the crystal homogeneity by X-Ray diffraction (XRD), optical microscopy, atomic force microscopy (AFM), 3D atom probe, and transmission electron microscopy.

**Figure 1:**
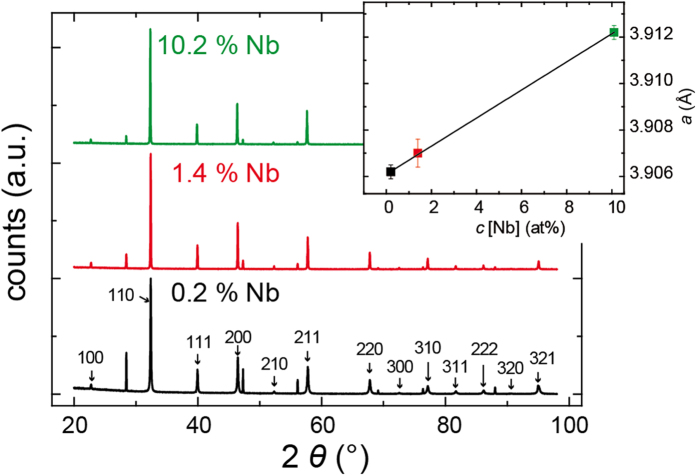
Powder diffraction pattern of the crystals. The inset shows the lattice parameter as a function of donor concentration. Adapted from ref. 26.

In Fig. 1, the powder diffraction pattern of crystals with different Nb content obtained by XRD are shown. All three crystals were found to be free of secondary phases^26^ as can also be extracted regarding Rocking curve measurements of the (100) peak discussed in Supplementary Information II. The pattern corresponds to the cubic perovskite structure with the space group Pm3m. Using Rietveld refinement, the lattice parameter was calculated and is shown in the inset of Fig. 1. It increased with the Nb concentration, which can be understood by taking into account that the Nb substituting the Ti has a higher atomic radius leading to an expansion of the unit cell according to Vegard´s law. Furthermore, we can conclude from the linear dependence between donor concentration and lattice parameter that a statistical Nb distribution is present in the Verneuil-grown crystals, which is supported by the fact that the solubility limit of Nb in SrTiO_3_ ceramics was reported to be above 40% being much higher than the Nb concentrations in the crystals investigated here^28^. The results fit well into the picture of an ideal solid solution Sr(Ti,Nb)O_3_ with statistically random distributed ions on the B-site of the perovskite lattice, which can be explained by the very similar radii of Ti and Nb ions in the SrTiO_3_ lattice^29^. A similar situation is reported for trivalent La ions incorporated as donors in SrTiO_3_ by substituting Sr ions. Here, Vegard´s law is obeyed in the entire solid solution range (Sr_1-*x*_La_*x*_)TiO_3_ for *x* = 0–1^30^. At approx. *x* = 0.5, superstructure lines have been observed^30^. Synthesis under oxidizing conditions needs to account for cation vacancies in the stoichiometry^31^. Despite these results the question remains if there are inhomogeneities on the submicron scale in the donor ion distribution which slip through the resolution of the XRD analysis.

**Figure 2:**
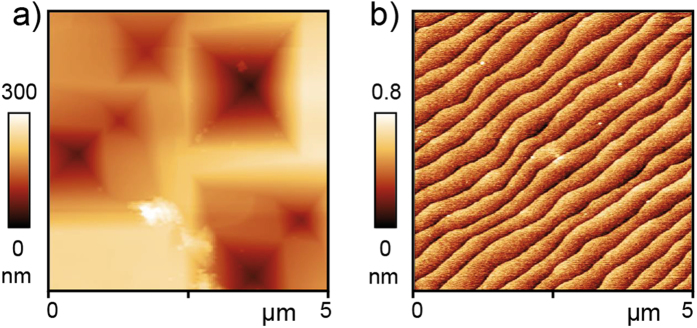
AFM analysis on a crystal doped with 1.4 at% Nb after BHF etching. (**a**) Topography of after 10 min BHF etching. Adapted from ref. 26. (**b**) Topography after wet etching for 225 s in BHF and annealing at 950 °C in air resulting in an atomically flat TiO_2_-terminated surface.

In order to investigate the density of dislocations, the etch pit method was used. The crystal doped with 1.4 at% Nb was heated in a buffered hydrofluoric acid (BHF) at a temperature of 90 °C. Under these conditions, a preferential etching at the core of dislocations is induced resulting in the evolution of etch pits which can thus be used as a marker of the dislocations. The investigation of the etched surface by optical microscopy revealed bundles of dislocations and agglomeration along striped structures illustrating that significant inhomogeneity with respect to extended defects is present in Verneuil-grown crystals^26^. Beside these dislocations bundles, a random distribution of etch pits with a density of approx. 6∙10^6^/cm^2^ was detected. Additional scanning electron images of the etched surface are shown in Supplementary Figure S4. Using AFM (Fig. 2a) we found that beside the larger etch pits visible in the optical images also smaller pits had evolved revealing a total density of dislocations of 10^8^/cm^2^ being in agreement with earlier reports^19^,^20^. It has to be kept in mind that the etching method cannot only be used to mark the dislocations by creating etch pits. Using a wet etching method involving rinsing the crystals in water, short BHF etching and annealing under oxidizing conditions, atomically flat TiO_2_-terminated surfaces can be generated (Fig. 2b), which are particularly utilized for the deposition of functional epitaxial thin films on a Verneuil-grown SrTiO_3_:Nb crystal acting as electronically conducting substrate^32^,^33^. Here, approx. 400 nm wide terraces of one unit cell height are obtained.

Furthermore, we analysed the quality of the crystals using electron backscatter diffraction (EBSD) shown as Supplementary Information III. We detected only regular stripes in the diffraction maps indicating local misorientations below 0.3° that we interpret as shear bands with enhanced dislocation density but no grains with low angle grain boundaries were found showing that the investigated samples can be regarded as single crystalline.

**Figure 3:**
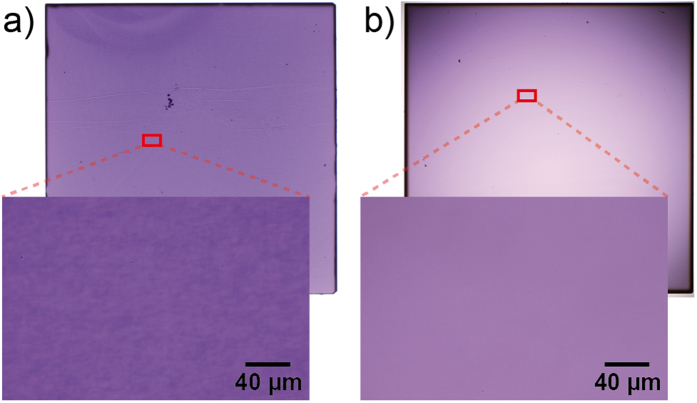
Optical microscopy of Nb-doped double sided polished SrTiO_3_ single crystals of boules from different production runs with a dimension of 5 mm × 5 mm × 0.1 mm in transmission light mode. (**a**,**b**) crystals from two different suppliers.

As described in our earlier work^24^, imperfections of Verneuil-grown crystals can already be seen in some samples with the naked eye when cutting the crystal boule in slices with thicknesses below 0.5 mm. In Fig. 3, photographs of 100 μm thick double-side polished Nb-doped crystals are shown. The crystal doped with 1.4 at% Nb (Fig. 3a) exhibits a variety of slight growth defects. Even in the most homogeneous regions, slight variations in the colour on the microscale with typical spatial frequency in the order of 5 to 20 μm were detected indicating via the Beer-Lambert-law that the electronic structure with respect to the occupancy of the Ti^3+^ levels varies within the crystal. As a measure for the modulation of the optical transmissivity we analysed the luminance of the obtained image showing a standard deviation of approx. 4%. The modulation signature of these variations appears to be production dependent indicating tiny imperfections of the crystal growth process as their origin. In addition, spatial inhomogeneities of further optical phenomena such as the intensity of the Raman effect and the lifetime of the fluorescence have been found on crystals with comparable quality^24^,^25^ but they do not directly imply that an inhomogeneous Nb distribution is present in the crystal as discussed below. On the other hand, it is possible to purchase Verneuil grown Nb-doped (1 at%) single crystals of even higher quality from a different supplier in which these modulations of the optical transmissivity are below the detection limit (Fig. 3b). Presumably the slight differences in the homogeneity of the crystals shown in Fig. 3a,b are due to differences in the Verneuil process parameters used by the two different suppliers. Despite these distinct optical variations, the density of dislocations monitored by etch pit technique as described above was in the same range in both crystals shown in Fig. 3, indicating that there is no direct impact of the dislocations on the electrical properties.

Although Nb-doped SrTiO_3_ should show metallic conductivity even at low doping concentrations^11^, the as-received crystals appear to be highly insulating upon contact by metallic needles due to the evolution of an oxidized surface layer with slight Sr excess^26^. Regarding the redox-equation of Nb doping


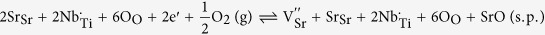


(here shown for two complete formula units of the perovskite lattice, for clarity) it can be seen that only under reducing conditions the positive charge of the Nb donors is compensated by electrons leading to metallic behaviour while under oxidizing conditions a compensation by Sr vacancies is active leading to the evolution of SrO as secondary phase^10^,^34^. This way, we can also understand the resistive switching of SrTiO_3_:Nb as an effect which takes place in the surface layer while the bulk constantly remains in the degenerate semiconducting metal-like state.

Investigating the surface layer using scanning probe techniques it can be seen that some degree of lateral inhomogeneity appears to be present on the surface even after annealing under UHV conditions at 500 °C to remove the physisorbates. In ref. 26 we reported about the resistive switching of a SrTiO_3_:Nb surface by conductive AFM. The area written into the low resistive state was also inhomogeneous showing conducting clusters with diameters of 20–50 nm. While comparable inhomogeneous surface conductivity on the nanoscale was found on all conducting oxides that we have investigated so far, the uncovering of the nature of this effect is still a challenging task for future research. Comparing the conductive AFM resistive switching data of Nb-doped crystals with undoped SrTiO_3_, where only small conducting and switchable spots related to dislocations embedded in an otherwise less conductive matrix were found^8^, we can conclude that upon doping the mechanism of electronic transport in the surface layer changes to the more homogeneous cluster-like conductance and that dislocations do not play an important role for the electronic transport anymore. Moreover, it was concluded by Chen *et al*. that dislocations could even decrease the electronic transport locally close to dislocations by trapping of charges in donor-doped SrTiO_3_^35^. A detailed discussion of the electronic transport along dislocations in doped and undoped SrTiO_3_ is presented in Supplementary Information V.

**Figure 4:**
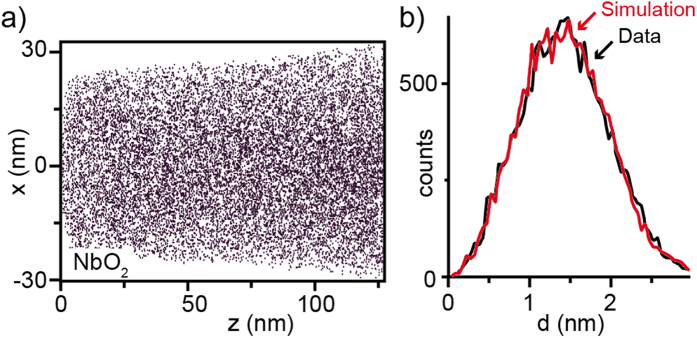
3D atom probe analysis. (**a**) Map of the Nb distribution (1.4 at% Nb) adapted from ref. 26. (**b**) Histogram of the nearest neighbour distribution of the detected NbO_2_^+^ ions (Simulation by CAMECA, division of AMETEK Inc.).

Having seen that various inhomogeneities may be present in Verneuil-grown crystals we can now focus on the question whether these inhomogeneities are related to inhomogeneous donor concentration. While the linear increase of the lattice parameter with the Nb content measured by XRD already gave a first indication for a statistical distribution of the Nb donor in the crystal, direct support for this assumption was given by secondary ion mass spectroscopy (SIMS) and atom probe for the crystal doped with 1.4% Nb. Within the resolution limit, no Nb agglomeration effects on the microscale were detected by SIMS. On the nanoscale the distribution of the Nb dopants was measured using 3D atom probe^26^. A small tip was cut out of the crystal with a focused ion beam and a map based on the measured NbO_2_^+^ ions was generated as shown in Fig. 4a. The nearest neighbour distances of adjacent NbO_2_^+^ ions were extracted and plotted as a histogram, which is in good agreement with the simulated curve of a random distribution (Fig. 4b, Simulation by CAMECA, division of AMETEK Inc.). This finding is supported by investigations of the SrTiO_3_:Nb surface by scanning tunnelling microscopy (STM). As reported by Marshall *et al*. it was possible to map single subsurface Nb dopants that were statistically distributed^36^ and even upon annealing the sample at high temperatures no Nb segregation was observed.

**Figure 5:**
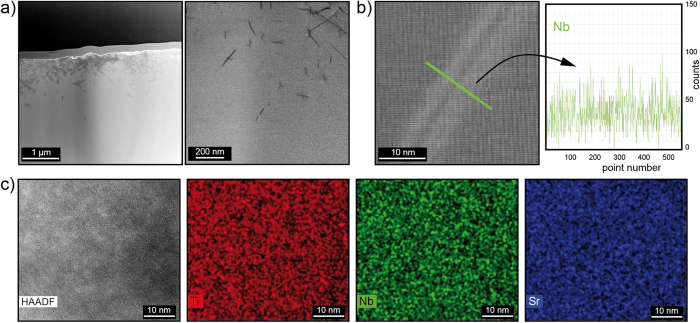
TEM imaging of a 10.2 at% Nb-doped SrTiO_3_ crystal. (**a**) TEM bright-field image of the dislocation-rich surface region. (**b**) HAADF image of a single dislocation and EDX Nb linescan measured across a dislocation: brighter contrasts are caused by strain fields, fine contrast variations are due to individual lattice planes. (**c**) HAADF image and corresponding EDX maps of Ti, Nb, and Sr.

To investigate the Nb distribution on the nano- and atomic scale we performed combined measurements by TEM and EDX. We chose a crystal doped with 10.2 at% Nb which was still single crystalline (cf. Fig. 1) but the density measured by Archimedes method was lower by approx. 4% than the theoretical density derived from the XRD measurement indicating the presence of growth defects such as voids. Also carbon contamination and macroscopic Nb segregation was revealed by SIMS^25^. Hence we assumed that if an inhomogeneous Nb distribution on the nanoscale existed, the probability of finding it would be the highest in these low-quality crystals doped with 10.2 at% Nb. For the TEM investigations, a part of the sample with minimum macroscopic Nb segregation was chosen and thinned by focused ion beam and subsequent Ar ion milling resulting in a specimen with a gradual thickness variation between 30 nm and more than hundred nanometres. Figure 5a displays a cross section, where the original surface of the sample is seen to exhibit a considerable roughness due to the mechanical cutting and polishing procedure of the single crystal. Close to the surface seen in the upper part (Fig. 5a left) a high density of 10^12^/cm^2^ dislocations is identified by their dark contrast lines in the bright field TEM image, whereas the regions farther away from the surface is apparently dislocation-free, i.e. the dislocation density is below 10^5^/cm^2^. Hence, the dislocations are induced by mechanical treatment of the single crystal and are not formed by the TEM specimen preparation procedure, since a homogenous distribution of dislocations would be expected in this case. In order to identify a potential Nb segregation to the dislocation cores by EDX measurements, a single dislocation about 2 μm away from the original surface was chosen, which we assume to be a grown-in “bulk” dislocation. Figure 5b displays a high-angle annular dark field (HAADF) image, where the strain fields surrounding the dislocation give rise to brighter contrast and the dislocation line shows lower intensity with respect to its vicinity. Of course, a dislocation, which would be “end-on”, i.e. extending perpendicular to the TEM sample’s surface, would be better suited for such an investigation, because a possible segregation would give rise to a larger signal in the EDX spectra. However, since the density of grown-in dislocations is very low, we had to live with a dislocation being inclined to the TEM foil. Since the SuperX EDX detector used for this measurement is highly sensitive to small concentration fluctuations and allows for detection well below 1 wt%^37^ a severe Nb segregation should give a distinct signal. However, within the detection limit, no evidence for an Nb segregation could be found along a line scan shown in green perpendicular to the dislocation. (The signal shown in the graph was obtained by summing the counts parallel to the dislocation to improve the signal to noise ratio.) The same holds for the Sr and Ti signals (not shown here), which remain constant across the dislocation line. Also in areas of large dislocation density no enhancement of the Nb concentration was detectable. Hence, we can exclude that a distinct Nb agglomeration is present within the dislocation cores which would in turn influence the macroscopic electrical properties of the sample significantly. In a thinner, dislocation-free region of the specimen, dark-field images reveal slight contrast changes on the nanometre scale. In order to investigate if this contrast feature is caused by Nb agglomeration, EDX elemental maps have been recorded. However, in the corresponding EDX maps of Ti, Nb, and Sr shown in Fig. 5c no evidence for a variation in composition on a length scale of 5 nm was found indicating that even in the defective, highly doped SrTiO_3_ crystal, in which macroscopic defects were induced kinetically due to non-perfect conditions during crystal growth, a random Nb distribution on the nanoscale is present.

## Conclusions

Using XRD, 3D atom probe analysis, optical microscopy, and high-resolution TEM with HAADF imaging and EDX mapping, we have investigated the distribution of Nb atoms in Verneuil grown SrTiO_3_ single crystals with Nb donor doping in the range from 0.2 at% to 10.2 at% Nb. While it is known that the quality of SrTiO_3_:Nb single crystals depends strongly on the conditions during growth process, the following conclusions can be drawn from our results:

1. In crystals doped with 1.4 at% Nb, that are commonly used as conducting substrate for epitaxial thin film deposition, a statistically random Nb distribution was found on the macro-, micro-, and nano-/atomic scale.
2. Slight variations of the electronic structure were seen in some specimen by investigations of the optical properties indicating a slightly inhomogeneous distribution of the Ti^3+^ states on the microscale even for high-quality Verneuil grown crystals. These inhomogeneities can be regarded as growth fluctuations and presumably arise from internal stress fields.
3. In addition, we investigated a higher doped crystal (10.2 at% Nb) with lower quality. Even in this defective crystal, a statistical Nb distribution on the nanoscale was found.
4. In particular, no Nb segregation at dislocations was detected. This is consistent with our previous conductive AFM studies which showed that dislocations do not play an important role in supporting the electronic transport in Nb:SrTiO_3_ crystals. In contrast, it was found that in particular in crystals with higher Nb doping extended defects such as dislocations^35^,^38^ and grain boundaries^10^,^39^ may trap electrons in donor doped SrTiO_3_ and, hence, reduce the electronic conductivity locally.

We conclude that there is no driving force for Nb agglomeration and the observed inhomogeneities of real crystals are artefacts of the growth process. Even if there are distinct variations in the macroscopic Nb distribution due to faults in the growth process, the critical density of delocalized charge carriers needed for the insulator-metal transition can be expected to be always achieved in the whole crystal^11^. Hence, a homogeneous metallic conductivity is present in the bulk of Nb-doped SrTiO_3_. Regarding the suitability of this material for applications we can conclude that in most cases the use of Verneuil-grown crystals is a very acceptable trade-off between quality and cost e.g. when using them as conducting substrates. If extremely homogeneous optical or electronic properties are needed, appropriate single crystals should be selected.

## Methods

Commercially available epi-polished SrTiO_3_:Nb (100) single crystals with doping concentration of 0.2, 1.0, 1.4 and 10.1 at% purchased from Crystec, Mateck, and SurfaceNet were investigated. Powder X-ray diffraction measurements were conducted on a STOE instrument using a Si reference in order to determine the lattice parameter precisely. AFM measurements were conducted on a JEOL instrument in tapping mode using a Si cantilever. Optical microscopy images were obtained by a Zeiss Axio A1 microscope in transmission light mode equipped with a 100 W LED light source adjusted to approx. 60%. Atom probe tomography was performed on needles cut out of the single crystals by FIB on a CAMECA LEAP4000 X HR instrument with UV-laser. TEM/EDX measurements were performed using an aberration corrected FEI Titan G2 80–200 transmission electron microscope equipped with a Super-X EDX detector. EDX maps have been recorded for 10 min with a lateral resolution of about 0.2 nm in 20–50 nm thick specimen areas. Simultaneously a HAADF image was acquired.

## Additional Information

**How to cite this article**: Rodenbücher, C. *et al*. Homogeneity and variation of donor doping in Verneuil-grown SrTiO_3_:Nb single crystals. *Sci. Rep.* **6**, 32250; doi: 10.1038/srep32250 (2016).

## Supplementary Material

Supplementary Information
