# Supplementary material for: Homogeneity and variation of donor doping in Verneuil-grown SrTiO3:Nb single crystals
Source: Sci Rep. 2016 Aug 31;6:32250. doi: 10.1038/srep32250 (PMC5006055; doi:10.1038/srep32250)
Supplement: Supplementary Information [file srep32250-s1.pdf]

Supplementary information

to

Homogeneity and variation of donor doping in Verneuil-grown $SrTiO_3$:Nb single crystals

C. Rodenbücher, M. Luysberg, A. Schwedt, V. Havel,

F. Gunkel, J. Mayer, and R. Waser

I. Photograph of the crystal

In order to illustrate the typical size and shape of the investigated samples, we show a macro photograph of an as-received crystal of the size of 5 x 5 x 0.1 mm^3 in Fig. S1. In the background, a cent coin can be seen for comparison. Both sides of the single $SrTi_{0.99}Nb_{0.01}O_3$ crystal were epi-polished resulting in a specular surface. The crystal had a deep purple colour due to the donor doping and was slightly translucent. On this macroscopic scale, no significant inhomogeneities cracks or impurities could be detected with the naked eye showing that the preparation of the crystal was performed with sufficient accuracy by the crystal growers.

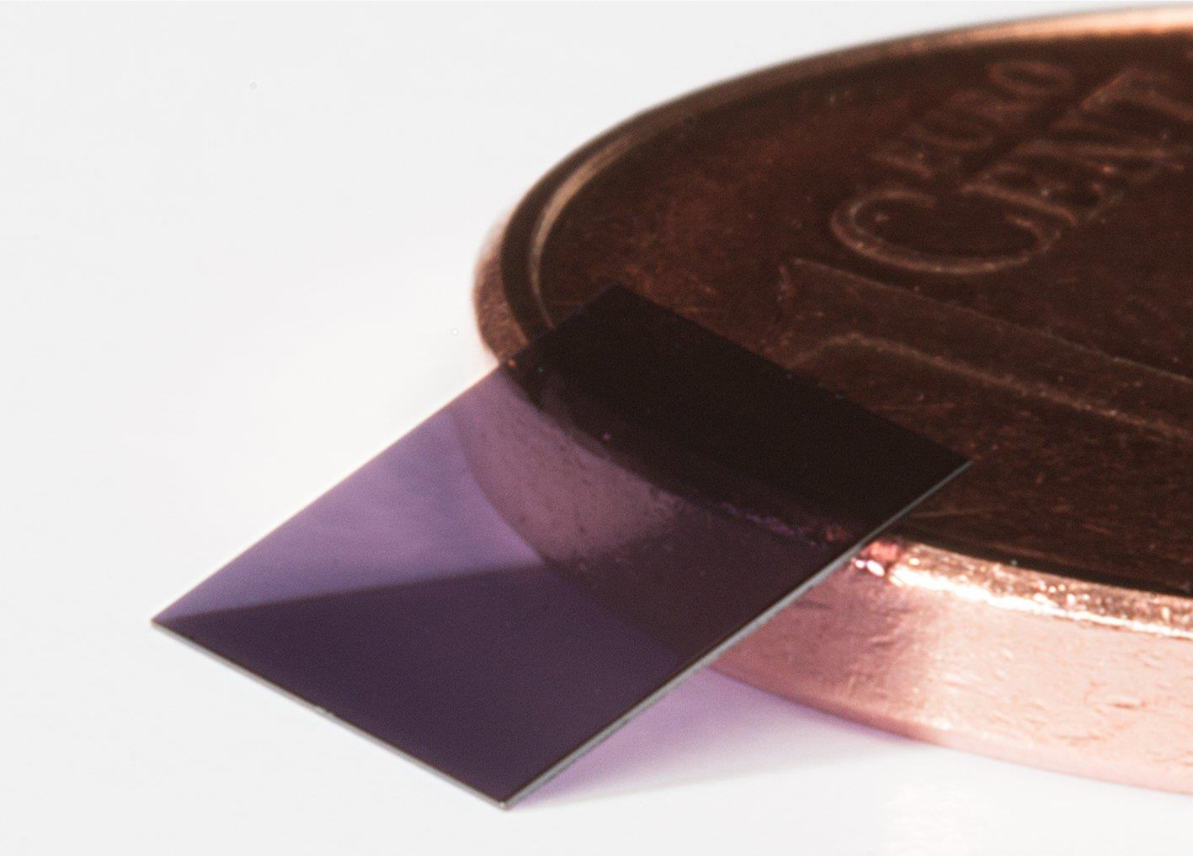

Fig. S1. Macro photograph of a 5 x 5 x 0.1 mm^3 Nb-doped $SrTiO_3$ crystal.
Moderate image processing involving overall brightness and contrast adjustment was applied.

II. XRD Rocking curve analysis

To investigate the homogeneity with respect to crystal structure, the crystals were investigated by Rocking curve analysis. After measuring XRD 2θ scans revealing a perovskite structure in the space group Pm3m free of secondary phases in agreement to the powder diffraction measurements shown in the main text, the (100) reflection was selected for Rocking curve measurements and the full width half maximum (FWHM) was calculated (Fig. S2). Two crystals from different suppliers doped with 1.0 % Nb (a) and 1.4 % Nb (b) were investigated. As expected for Verneuil-grown crystals both were not perfectly single-crystalline and did not show the typical 'one' (symmetric) reflection. Especially the Rocking curve of sample (b) is split. The comparison however shows that sample (a) seems to be of higher quality than sample (b) because of the 'nearly typical' Rocking curve with a lower FWHM. This demonstrates that the quality depends strongly on the conditions during crystal growth but that it is in principle possible to produce crystals with acceptable quality with the fast and efficient Verneuil method.

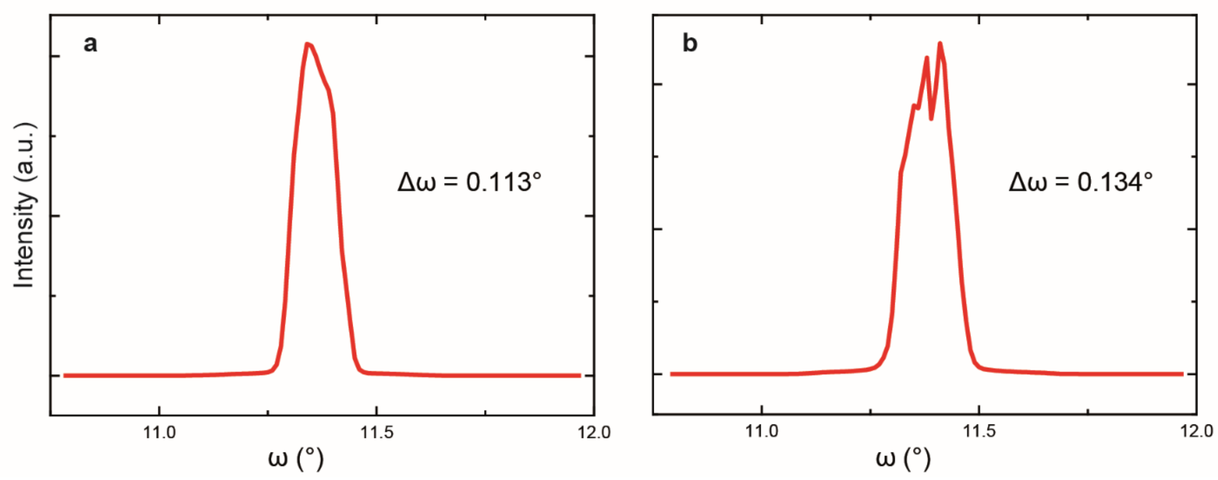

Fig. S2. Rocking curve measurement of the (100) reflection of $SrTi_{1-x}Nb_xO_3$ crystals of two different suppliers, with x = 1.0 % (a) and x = 1.4 % (b).

III. EBSD mapping

In order to analyse the quality of the crystals regarding crystallographic orientation, we have executed additional electron backscatter diffraction (EBSD) studies using a Hikari camera by EDAX-TSL attached to a JSM 7000F SEM by JEOL. For control experiments, the measurements were repeated for various orientations of the crystal with respect to the detector. In Fig. S3, the corresponding Kernel Orientation Maps for the $SrTi_{1-x}Nb_xO_3$ single crystals with x = 1.0 % and x = 1.4 % are shown. The measurements have been performed at 20 keV electron energy and approx. 30 nA beam current and cover an area of 1 mm^2 with a step size of 1µm. We observe regular patterns of stripes, at which accumulated slight misorientiations of up to 0.3° are suddenly reduced, and the crystal orientation returns to its initial values. These stripes are approx. 300 µm apart from each other. Due to their shape and pattern, they are clearly not caused by subgrains or any mosaicity. The fact that we were able to detect misorientations far below 1° demonstrates that the single crystals were free of larger structural imperfections such as grain boundaries across the entire surface of the 10 mm x10 mm samples. In agreement with the Rocking curve analysis these results reveal that the quality of crystal (a) is higher than that of crystal (b). The observed bands of slight misorientation must be caused by processes after the growth of the crystals, e.g. stress caused by the annealing, or else probably resulting in the evolution of ordered shear bands with locally enhanced dislocation density.

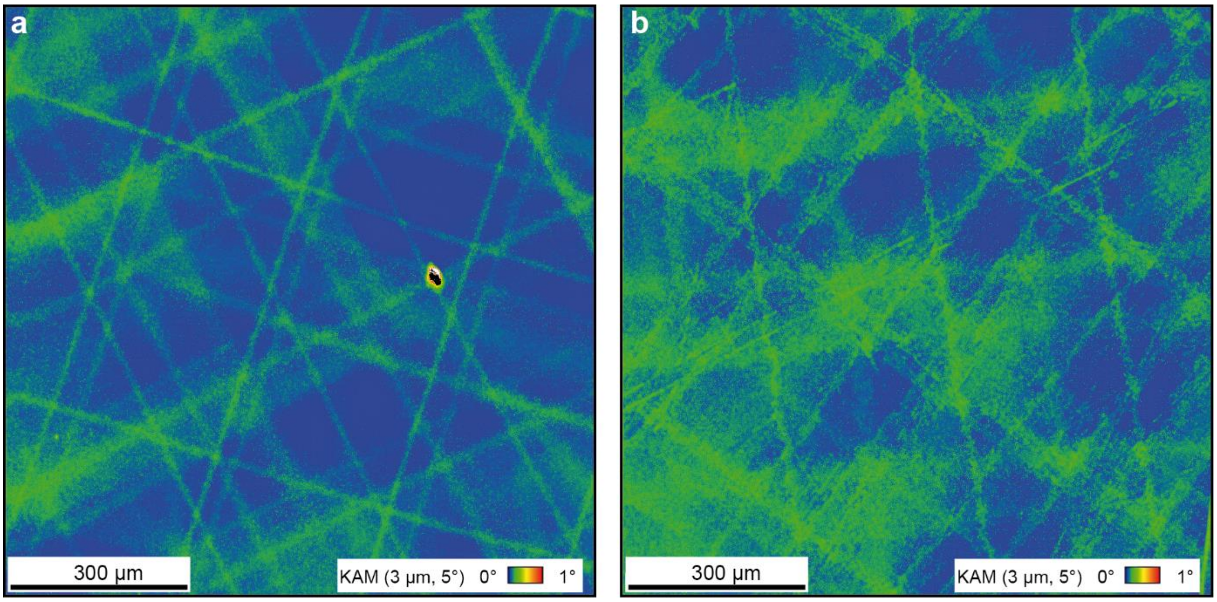

Fig. S3. EBSD Kernel Orientation Maps of $SrTi_{1-x}Nb_xO_3$ crystals of two different suppliers, with x = 1.0 % (a) and x = 1.4 % (b).

IV. Etch-pits analysis

The distribution of dislocation in the crystal was analysed using etch-pit technique as described in the main text of our paper. Here we show mappings of the etch pit distribution on the larger scale obtained after HF etching for 5 minutes using the scanning electron microscope (SEM). A etch pit density of the investigated crystal (1.0 at% Nb) of $(2.5 \pm 0.4)\cdot 10^6/\text{cm}^2$ was estimated. In Fig. S4 it can be seen that the distribution of etch pits is rather random and only in a few selected areas linear arrangements of etch pits aligned along the crystal axes which indicates an ordering of dislocations can be observed. Hence, we could not find a direct correlation between the linear structures observed by EBSD to the distribution of dislocations. Here, we have to keep in mind that during cutting and polishing of the samples by the crystal growers, the surface layers were altered significantly leading to the evolution of additional dislocations which induced distortion and rearrangement. Hence this could be a reason why we did not observe such a regular pattern in the distribution of dislocations as expected from EBSD. However regarding the magnification in Fig. S4b it can be seen that the etch pit distribution is not completely random but that in many cases two or more etch pits appear very close to each other, which is in agreement to the AFM results shown in Fig. 2.

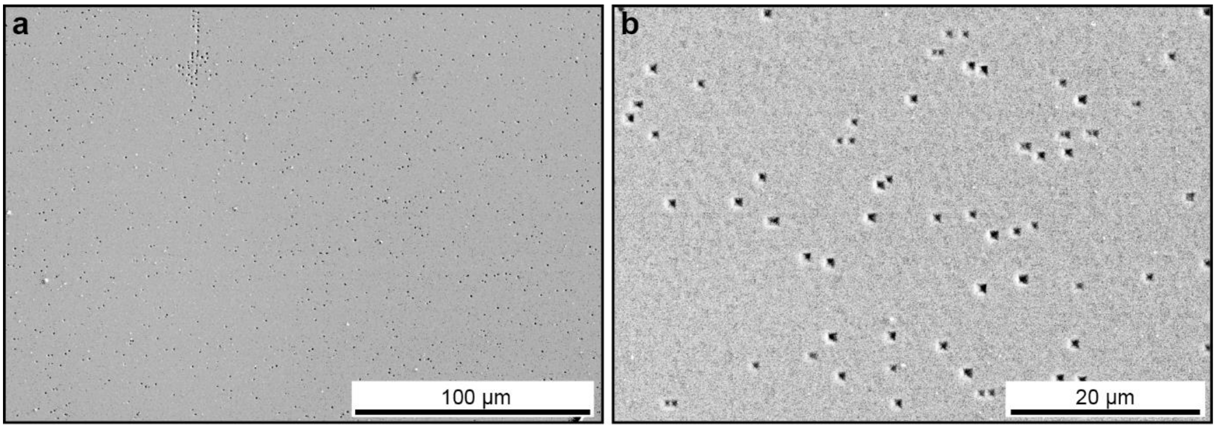

Fig. S4. SEM images of the distribution of etch pits on the surface of a crystal doped with 1.0 at% Nb after etching for 5 min in hydrofluoric acid.

V. Impact of extended defects on the electronic conductivity of undoped and donor-doped $SrTiO_3$ crystals

In the main text we briefly report about the low impact of dislocations on the electronic transport properties of electron compensated donor-doped $SrTiO_3$ single crystals which is in contrast to the effect of dislocations in undoped $SrTiO_3$ crystals. Here we provide background information about these statements.

In undoped $SrTiO_3$ which is always very slightly acceptor-doped $SrTiO_3$ because of (a low concentration of) Sr vacancies originating from the Schottky equilibrium during crystal formation and traces of acceptor-type impurities, there are O vacancies acting as donors compensating the acceptors[1, 2]. Annealing under oxidizing and moderately reducing atmospheres leads to an electrically insulating titanate. In the core of extended defects such as grain boundaries[3] and dislocations[4, 5], there is a higher concentration of O vacancies[6] and these are less mobile (i. e. structurally fixed) compared to highly mobile O vacancies in the bulk lattice[7-9]. Due to the donor character of O vacancies in $SrTiO_3$, annealing under moderately reducing conditions leads to the onset of an electron conduction along dislocation. Presumably this is the origin of the electron conductivity spots at the exits of dislocations at the surface of $SrTiO_3$ crystals which can be detected and memristively switched by local-conduction AFM [9, 10].

In donor-doped $SrTiO_3$ in the electron compensated regime, we observe a somewhat opposite situation. In contrast to highly mobile O vacancies, Sr vacancies show an extremely low diffusion coefficient in the bulk lattice of $SrTiO_3$[11]. As a consequence, oxidizing annealing of donor doped $SrTiO_3$ (and isostructural $BaTiO_3$) proceeds only very slowly, starting from surface[11], grain boundaries[12], or dislocations[13]. Sr vacancies which act as acceptors compensate the donors locally and, in addition, there is a space charge depletion zone of few nanometers. Hence, dislocations in donor-doped $SrTiO_3$ may be tiny insulating “wires” in an electronically conducting matrix. However, on the density levels reported here, they have no significant effect on the overall conductivity (in contrast to grain boundaries), because they are “by-passed” by the electrons in the bulk.

VI. EDX analysis

On the macroscale, the homogeneity of the crystals was analysed by electron dispersive X-ray spectroscopy (EDX) in combination with scanning electron microscopy (SEM). In different areas of a crystal doped with 1.0 at%, linescans were performed and the concentration of the elements was measured as shown exemplarily in Fig. S6 for a distance of 1 mm. It can be seen that the concentration of all elements did not vary with the position (the variations in the Nb signal can be attributed to statistical fluctuations of the measurement due to the low signal-noise ratio). This proves that the crystals that are commonly used as substrates for the growth of functional thin films can be regarded as homogeneous on the macroscale.

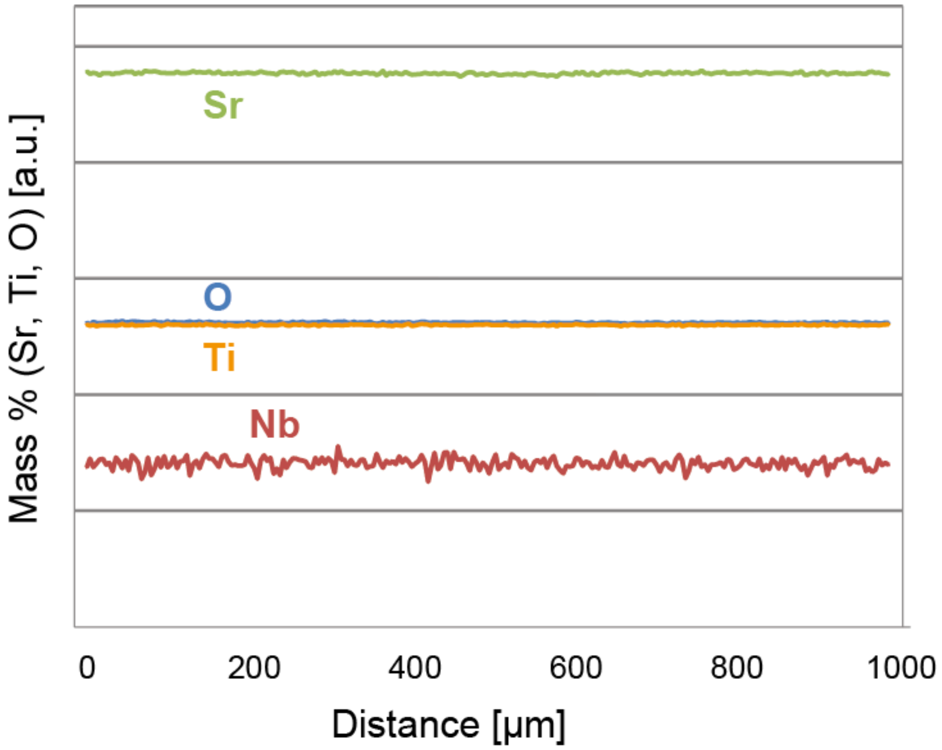

Fig. S5. EDX linescan of a $SrTiO_3$ crystal doped with 0.5 wt% Nb.

References

1. Smyth, D. M. Defect structure in perovskite titanates. *Current Opinion in Solid State & Materials Science* **1**, 692-697 (1996).

2. Merkle, R. & Maier, J. How is oxygen incorporated into oxides? A comprehensive kinetic study of a simple solid-state reaction with $SrTiO_3$ as a model material. *Angew. Chem.-Int. Edit.* **47**, 3874-3894 (2008).

3. Waser, R. & Hagenbeck, R. Grain boundaries in dielectric and mixed-conducting ceramics. *Acta Mater.* **48**, 797-825 (2000).

4. Zhang, Z., Sigle, W. & Rühle, M. Atomic and electronic characterization of the a[100] dislocation core in $SrTiO_3$. *Physical Review B (Condensed Matter and Materials Physics)* **66**, 94108-1-8 (2002).

5. Du, H. *et al.* Atomic structure and chemistry of dislocation cores at low-angle tilt grain boundary in $SrTiO_3$ bicrystals. *Acta Mater.* **89**, 344-351 (2015).

6. McIntyre, Paul C. Equilibrium Point Defect and Electronic Carrier Distributions near Interfaces in Acceptor-Doped Strontium Titanate. *J. Am. Ceram. Soc.* **83**, 1129-36 (2000).

7. De Souza, R. A. Oxygen Diffusion in $SrTiO_3$ and Related Perovskite Oxides. *Advanced Functional Materials* **25**, 6326-42 (2015).

8. Marrocchelli, D., Sun, L. & Yildiz, B. Dislocations in $SrTiO_3$: Easy To Reduce but Not so Fast for Oxygen Transport. *J. Am. Chem. Soc.* **137**, 4735-4748 (2015).

9. Waldow, S. P. & De Souza, R. A. Computational Study of Oxygen Diffusion along a[100] Dislocations in the Perovskite Oxide $SrTiO_3$. *ACS Applied Materials Interfaces* **8**, 12246-56 (2016).

10. Szot, K., Speier, W., Bihlmayer, G. & Waser, R. Switching the electrical resistance of individual dislocations in single-crystalline $SrTiO_3$. *Nat. Mater.* **5**, 312-320 (2006).

11. Meyer, R., Waser, R., Helmbold, J. & Borchardt, G. Observation of vacancy defect migration in the cation sublattice of complex oxides by ^{18}O tracer experiments. *Phys. Rev. Lett.* **90**, 105901/1-4 (2003).

12. Daniels, J., Hardtl, K. H., Hennings, D. & Wernicke, R. Defect Chemistry And Electrical-Conductivity Of Doped Barium-Titanate Ceramics. *Philips Res. Rep.* **31**, 487-488 (1976).

13. Chen, J., Sekiguchi, T., Li, J. & Ito, S. Investigation of dislocations in Nb-doped (100) $SrTiO_3$ single crystals and their impacts on resistive switching. *Superlattices and Microstructures* (2016).
